# Overexpression of G protein-coupled receptor GPR87 promotes pancreatic cancer aggressiveness and activates NF-κB signaling pathway

**DOI:** 10.1186/s12943-017-0627-6

**Published:** 2017-03-14

**Authors:** Li Wang, Wei Zhou, Yunfeng Zhong, Yongbao Huo, Ping Fan, Sudong Zhan, Jun Xiao, Xin Jin, Shanmiao Gou, Tao Yin, Heshui Wu, Tao Liu

**Affiliations:** 1 Department of General Surgery, Union Hospital, Tongji Medical College, Huazhong University of Science and Technology, Wuhan, 430022 People’s Republic of China; 2 Department of Pancreatic Surgery, Union Hospital, Tongji Medical College, Huazhong University of Science and Technology, Wuhan, 430022 People’s Republic of China; 3 Department of Digestive Surgical Oncology, Cancer Center, Union Hospital, Tongji Medical College, Huazhong University of Science and Technology, Wuhan, 430022 People’s Republic of China

**Keywords:** GPR87, Pancreatic cancer, NF-κB signaling, Apoptosis, Tumorigenicity

## Abstract

**Background:**

Pancreatic cancer is a highly lethal disease and has the worst prognosis of any major malignancy. G protein-coupled receptor GPR87 is reported to be overexpressed in multiple cancers. The clinical significance and biological role of GPR87 in pancreatic cancer, however, remain to be established.

**Methods:**

GPR87 expression in pancreatic cancer cell lines, paired patient tissues were determined using western blotting and Real-time PCR. Ninety-six human pancreatic cancer tissue samples were analyzed by immunochemistry (IHC) to investigate the association between GPR87 expression and the clinicopathological characteristics of pancreatic cancer. Functional assays, such as anchorage-independent growth, chicken chorioallantoic membrane (CAM) assay, transwell matrix penetration assay, and Annexin V-FITC and PI staining and a xenograft tumor model were used to determine the oncogenic role of GPR87 in human pancreatic cancer progression. The effect of GPR87 on NF-κB signaling pathway was further investigated using the luciferase reporter assays, and by detection of the NF-κB signaling downstream genes.

**Results:**

Herein, we reported that GPR87 was markedly overexpressed in pancreatic cancer cells and clinical tissues. Immunohistochemical analysis showed that the expression of GPR87 significantly correlated with patients’ clinicopathologic features, including clinical stage and tumor-nodule-metastasis (TNM) classification. Pancreatic cancer patients with higher levels of GPR87 expression had shorter overall survival compared to patients with lower GPR87 levels. We gained valuable insights into the mechanism of GPR87 expression in pancreatic cancer cells by demonstrating that overexpressing GPR87 significantly enhanced, whereas silencing endogenous GPR87 inhibited, the proliferation, angiogenesis and increased resistance to gemcitabine-induced apoptosis of pancreatic cancer in vitro and tumorigenicity of pancreatic cancer cells in vivo. Finally, we demonstrated that GPR87 enhanced pancreatic cancer aggressiveness by activating NF-κB signaling pathway. Conclusions: Taken together, these findings suggest that GPR87 plays a critical oncogenic role in pancreatic cancer progression and highlight its potential as a target for pancreatic cancer therapy.

**Conclusions:**

Our findings suggest that GPR87 plays a critical oncogenic role in pancreatic cancer progression and highlight its potential as a target for pancreatic cancer therapy.

**Electronic supplementary material:**

The online version of this article (doi:10.1186/s12943-017-0627-6) contains supplementary material, which is available to authorized users.

## Background

Pancreatic cancer is one of the most aggressive and lethal malignancies and ranks as the eighth most common cause of death from cancer worldwide [1, 2]. Despite the wide application of surgical resection, chemotherapy and/or chemoradiotherapy, pancreatic cancer still has the worst prognosis of any major malignancy with a 5-year survival rate of <5% and a median survival of <6 months [3, 4]. Therefore, it will be of great clinical value to identify effective treatment strategies and explore the molecular mechanisms responsible for the pathogenesis of pancreatic cancer to improve the prognosis of pancreatic cancer patients.

Increasing evidence suggests that unsatisfactory therapeutic outcomes and the poor prognosis associated with pancreatic cancer are related to aberrantly activated signaling pathways, including NF-κB signaling [5, 6]. Constitutive activation of NF-κB signaling pathway plays an important role in the development and progression of pancreatic cancer and contributes to a malignant phenotype. Arlt et al. reported that constitutive NF-κB activity confers resistance to gemcitabine-induced cell death in pancreatic cancer, while inhibition of NF-κB strongly diminishes gemcitabine resistance [5]. Maier and colleagues showed that NF-κB activation contributes to epithelial-mesenchymal transition, migration and invasion of pancreatic carcinoma cells, while these malignant phenotypes are suppressed upon NF-κB inhibition [7]. It has been reported that activation of NF-κB signaling pathway also promotes pancreatic tumor angiogenesis [8] and metastasis [8, 9]. Conversely, inhibition of NF-κB signaling pathway increases the number of apoptotic pancreatic cells [10] and inhibiting constitutive NF-κB activity by expressing phosphorylation defective IκBα significantly suppresses pancreatic cancer cell tumorigenesis [11]. Therefore, identifying novel molecules that modulate the NF-κB signaling pathway could be important for pancreatic cancer therapy.

G protein-coupled receptor (GPCR) 87, a newly identified gene located on chromosome 3q24, encodes a protein that contains an extracellular N terminus, seven helices, three intracellular loops, three extracellular loops and an intracellular C terminus [12]. It is a cell surface GPCR that is overexpressed in various cancers and plays vital role in tumor cell survival [13, 14]. A previous study using GPCR-focused Affymetrix microarrays to examine the expression of 929 GPCR transcripts in tissue samples from 10 patients with lung squamous cell carcinoma identified GPR87 as one of five GPCRs significantly overexpressed in this disease [15]. NII and colleagues reported that overexpression of GPR87 in non-small cell lung carcinoma (NSCLC) is significantly correlated with poorer differentiation and that higher expression of GPR87 is significantly associated with poorer survival for patients with NSCLC [16]. Moreover, GPR87 is essential for p53-dependent cell survival of RKO and MCF7 cell lines in response to DNA damage induced by doxorubicin or camptothecin and may serve as a novel therapeutic target for cancer treatment and prevention [17]. Zhang et al. reported that GPR87 is overexpressed in bladder cancer and promotes cell proliferation in bladder cancer cells [18]. Importantly, overexpression of GPR87 upregulates expression of cancer stem cell marker CD133 and promotes the growth and metastasis of CD133^+^ cancer stem-like cells in hepatocellular carcinoma [19].

Herein, we reported that GPR87 expression was significantly upregulated in pancreatic cancer and clinical tissues, and was correlated with the clinical features of pancreatic cancer. Overexpression of GPR87 promotes proliferation, metastasis, angiogenesis and resistance to apoptosis induced by a chemotherapeutic agent in pancreatic cancer. Our findings suggest that GPR87 plays a critical oncogenic role in pancreatic cancer progression and highlight its potential as a target for pancreatic cancer therapy.

## Results

### GPR87 overexpression is correlated with pancreatic cancer progression and poor prognosis

**Fig. 1:**
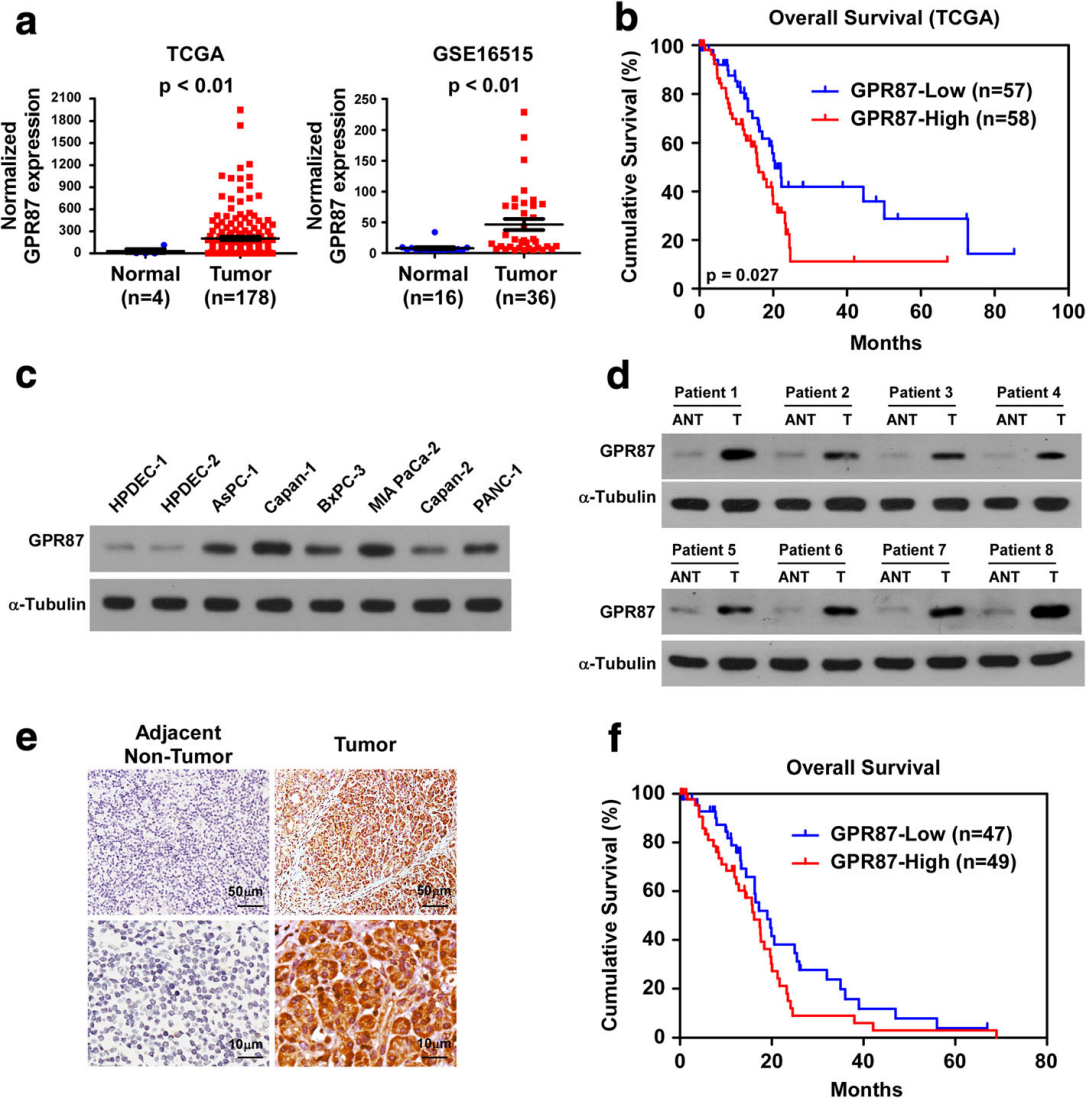
Overexpression of GPR87 correlates with pancreatic carcinoma progression and poor prognosis. **a**. Expression profile of GPR87 mRNA in primary pancreatic cancer tissues (*n* = 178) and normal pancreatic tissues (*n* = 4; *p* < 0.01; TCGA) (*left panel*); expression profile of GPR87 mRNA in primary pancreatic cancer tissues (*n* = 36) and normal pancreatic tissues (*n* = 16; *p* < 0.01; GSE16515) (*right panel*). **b**. Kaplan-Meier survival curves comparing pancreatic cancer patients with low and high GPR87 expression levels (*n* = 115; *p* < 0.027; TCGA). Higher and lower expression was based on median value of GPR87 mRNA. **c**. Western blot analysis of GPR87 expression in human pancreatic ductal epithelial cells (HPDECs) and six pancreatic cancer cell lines. **d**. Expression of GPR87, as determined by Western blot, in eight paired primary pancreatic cancer tissues (T) and the matched adjacent non-tumor tissues (ANT) from the same patient. α-tubulin served as a loading control. **e**. IHC staining indicating GPR87 protein expression in human primary pancreatic cancer compared with adjacent pancreatic tissues. **f**. Kaplan–Meier analysis of overall survival stratified by low GPR87 expression (*n* = 47) and high GPR87 expression (*n* = 49). GPR87 upregulation was significantly correlated with shorter overall survival (*p* = 0.040)

By analyzing datasets from The Cancer Genome Atlas (TCGA) and GSE16515 (http://www.ebi.ac.uk/arrayexpress/experiments/E-GEOD-16515/?query=gse16515.), we found that GPR87 expression was significantly upregulated in primary pancreatic cancer tissues compared with normal pancreatic tissue (Fig. 1a). Kaplan-Meier survival analysis of data from TCGA revealed that pancreatic cancer patients with higher expression of GPR87 had shorter overall survival (Fig. 1b). Western Blot and real-time PCR was performed and showed that GPR87 expression was upregulated in the six pancreatic cancer cell lines compared with normal human pancreatic ductal epithelial cells (HPDECs; Fig. 1c and Additional file 1: Figure S1A). Consistent with these findings, comparative analysis revealed that GPR87 was markedly overexpressed in eight primary pancreatic cancer samples with matched adjacent normal pancreatic tissues (Fig. 1d and Additional file 1: Figure S1B). These findings confirmed the data obtained from TCGA and the results from cell lines, suggesting that GPR87 is overexpressed in human pancreatic cancer.

To determine the clinical relevance of GPR87 in pancreatic cancer, GPR87 was examined in 96 paraffin-embedded, archived pancreatic cancer tissues by immunohistochemistry (IHC; (Additional file 2: Table S1). As shown in Fig. 1e, GPR87 was significantly upregulated in pancreatic tumors compared with matched adjacent normal pancreatic tissues. Furthermore, GPR87 levels were correlated with clinical stage (*p* = 0.022), and TNM classification (T: *p* = 0.003, N: *p* = 0.001, M: *p* < 0.001) in patients with pancreatic cancer (Additional file 3: Table S2). Kaplan-Meier survival analysis revealed that higher expression of GPR87 correlated with shorter overall survival (Fig. 1f). Collectively, these findings suggest a potential association between GPR87 upregulation and progression of pancreatic cancer.

### Upregulation of GPR87 promotes the aggressiveness of pancreatic cancer in vitro

**Fig. 2:**
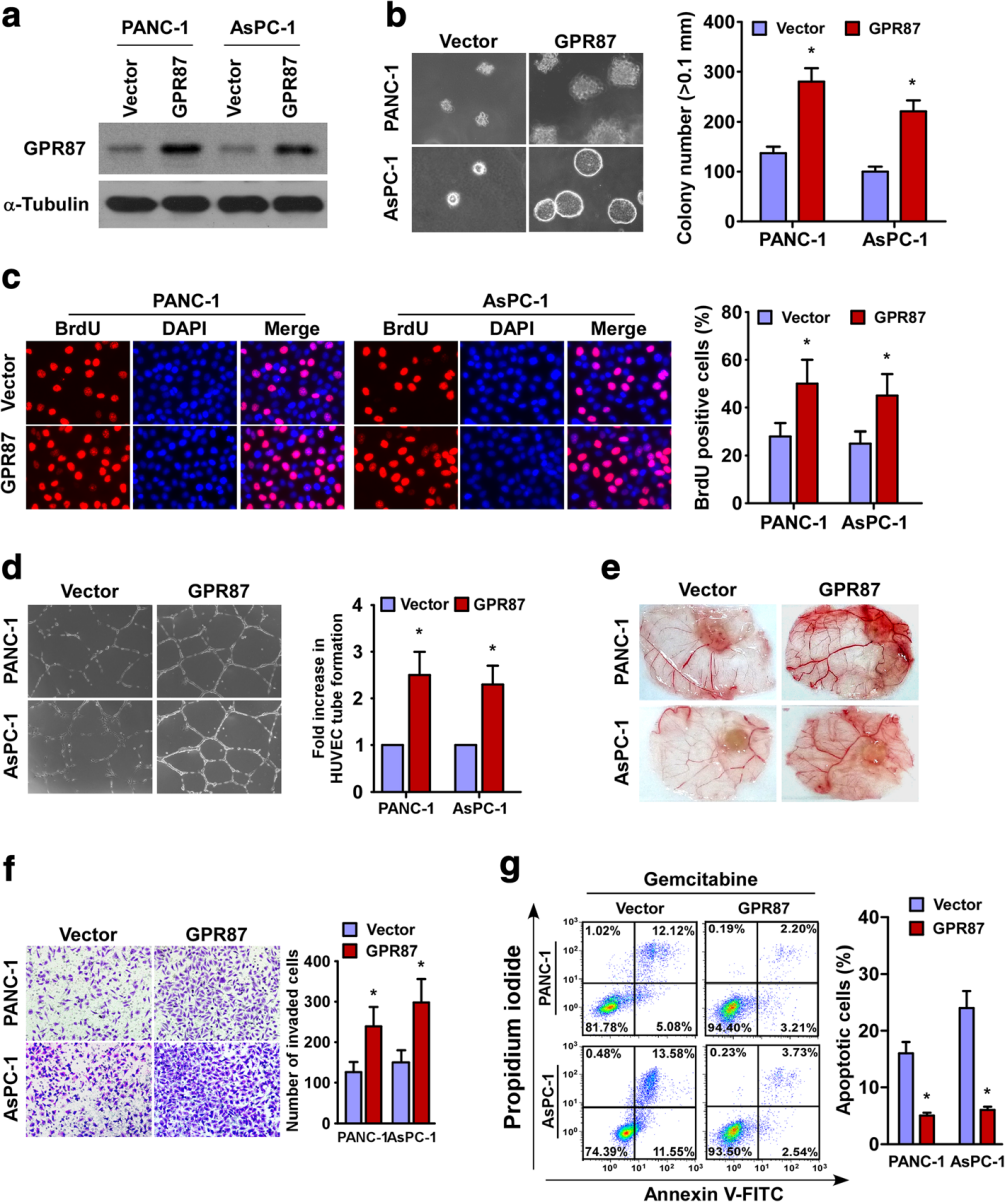
Upregulation of GPR87 expression promotes pancreatic cancer cell aggressiveness in vitro. **a**. Western blot of GPR87 expression in PANC-1 and AsPC-1 cells stably overexpressing GPR87. α-tubulin was used as a loading control. **b**. Representative images (*left panel*) and quantification (*right panel*) of colonies in an anchorage-independent growth assay. Colonies larger than 0.1 mm in diameter were scored. **c**. Representative micrographs (*left panel*) and quantification (right panel) of BrdU labeling in cells transfected with GPR87 or a vector control. **d**. Representative images (*left panel*) and quantification (*right panel*) of HUVECs cultured on matrigel-coated plates with conditioned medium from vector control or GPR87-transduced pancreatic cancer cells. **e**. Representative images of CAM blood vessels stimulated with conditioned medium from the indicated cells. **f**. Representative images (*upper panel*) and quantification (*lower panel*) of invading cells based on a transwell matrix penetration assay. **g**. Representative images (*left panel*) and quantification (*right panel*) of Annexin V-FITC and PI staining of PANC-1 and AsPC-1 cells overexpressing GPR87 treated with gemcitabine (1 μM) for 24 h. Each bar represents the mean ± SD of three independent experiments. * *p* < 0.05

To explore the biological role of GPR87 overexpression in pancreatic cancer progression, PANC-1 and AsPC-1 cell lines stably overexpressing GPR87 were established (Fig. 2a). We found that the anchorage-independent growth ability of GPR87-overexpressing pancreatic cancer cells dramatically augmented up to 2 folds compared to control cells (Fig. 2b). Furthermore, BrdU incorporation assay was performed and showed that only approximately 25 ~ 28% of control cells incorporated BrdU as compared with about 45 ~ 50% of GPR87-transduced cells, indicating that overexpressing GPR87 significantly increased the growth rate of pancreatic cells (Fig. 2b). In addition, the ability of pancreatic cancer cells to induce human umbilical vein endothelial cell (HUVEC) tube formation, as indicated by the drastic increased length of the completed tubes, and chicken chorioallantoic membrane (CAM) neovascularization, and invasive capability was markedly increased in the GPR87-overexpressing pancreatic cells (Fig. 2d-f). Moreover, overexpression of GPR87 also increased the resistance of pancreatic cancer cells to apoptosis induced by chemotherapeutic agents gemcitabine (Fig. 2g). Furthermore, we also found that overexpressing GPR87 or silencing GPR87 only resulted in slightly change of apoptotic rate of pancreatic cancer cells without any treatment (Additional file 4: Figure S2A). Taken together, these results suggest that GPR87 overexpression promotes the aggressiveness of pancreatic cancer cells in vitro.

### Silencing GPR87 suppresses the aggressiveness of pancreatic cancer cells in vitro

**Fig. 3:**
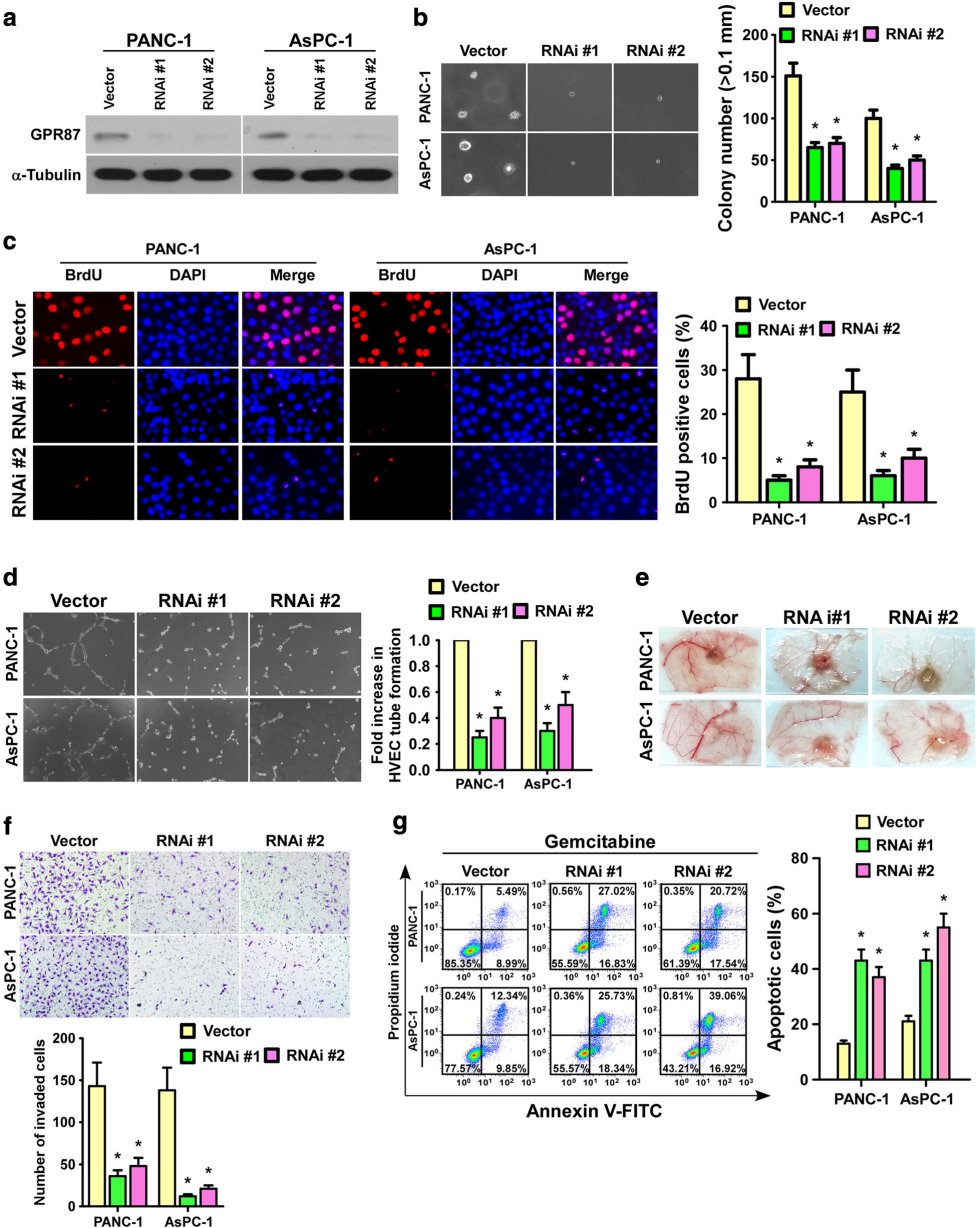
Downregulation of GPR87 decreases the aggressiveness of pancreatic cancer cells. **a**. Western blot analysis of GPR87 expression in PANC-1 and AsPC-1 cells following GPR87 silencing by RNAi. α-tubulin was used as a loading control. **b**. Representative images (*left panel*) and quantification (*right panel*) of colonies in an anchorage-independent growth assay. Colonies larger than 0.1 mm in diameter were scored. **c**. Representative micrographs (*left panel*) and quantification (*right panel*) of BrdU labeling in cells transfected with GPR87-RNAi or an RNAi-vector. **d**. Representative images (left panel) and quantification (*right panel*) of HUVECs cultured on matrigel-coated plates with conditioned medium from control and GPR87-RNAi pancreatic cancer cells. **e**. Representative images of CAM blood vessels stimulated with conditioned medium from the indicated cells. **f**. Representative images (*upper panel*) and quantification (*lower panel*) of invaded cells analyzed using a transwell matrix penetration assay. **g**. Representative images (*left panel*) and quantification (*right panel*) of Annexin V-FITC and PI staining of indicated cells treated with gemcitabine (1 μM) for 24 h. Each bar represents the mean ± SD of three independent experiments. * *p* < 0.05

Downregulation of GPR87 significantly inhibited proliferation of pancreatic cells (Fig. 3a-c), as shown nearly 2- to 2.5-fold decreased colonies and up to 2.5- to 5-fold reduced BrdU-positive cells in GPR87-silenced pancreatic cancer cells compared to control cells. In addition, silencing GPR87 decreased the invasive capacity of pancreatic cells as evaluated by the Transwell Cell invasion Matrigel Assay (Fig. 3d). Furthermore, HUVEC tube formation and CAM assays showed the abilities of pancreatic cancer cells to induce HUVEC tube formation and CAM neovascularization were inhibited in response to GPR87 knockdown (Fig. 3e-f). While Annexin V assay showed that the sensitivity of GPR87-silenced pancreatic cells to gemcitabine or Fluorouracil was drastically increased compared with control cells (Fig. 3g and Additional file 4: Figure S2B). Together, these data indicate that downregulation of GPR87 expression inhibits pancreatic cancer aggressiveness in vitro.

### Overexpression of GPR87 contributes to pancreatic cancer progression in vivo

**Fig. 4:**
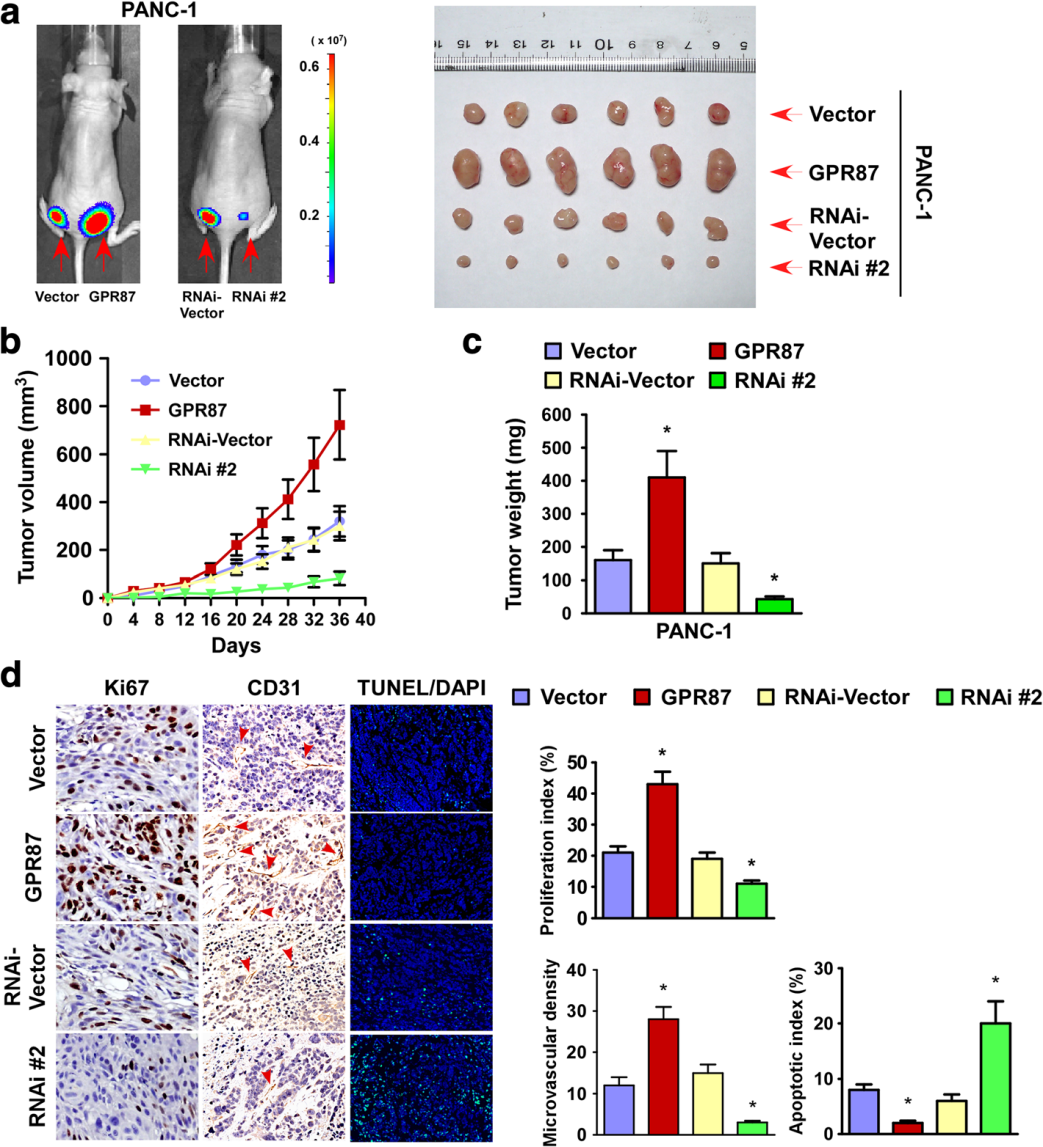
Overexpression of GPR87 contributes to pancreatic cancer progression in vivo. **a**. Representative images of tumor-bearing mice (*left*) and tumors from mice in each group (*right*). **b**. Tumor volumes were measured on the indicated days. **c**. Mean tumor weights. **d**. IHC staining demonstrating that overexpression of GPR87 induces while suppression of GPR87 inhibits the aggressive phenotype of pancreatic cancer cells in vivo, as indicated by the expression of Ki67 and CD31 as well as TUNEL-positive cells. * *p* < 0.05

To examine the biological effects of GPR87 on pancreatic cancer progression, a xenograft tumor model was used. As shown in Fig. 4a-c and Additional file 5: Figure S3, tumors formed by GPR87-overexpressing PANC-1 cells were larger and heavier than the tumors formed by control cells. Conversely, tumors formed by GPR87-silenced PANC-1 cells were smaller and had lower tumor weights than the control tumors. Furthermore, IHC analysis revealed that GPR87-overexpressing tumors showed higher percentages of Ki-67-positive cells, greater microvascular density (MVD) and fewer TUNEL-positive cells, whereas GPR87-silenced tumors displayed lower Ki-67 proliferation index and MVD and an increased percentage of TUNEL-positive apoptotic cells compared with control cells (Fig. 4d). Collectively, our findings emphasize the role of oncogenic GPR87 in pancreatic cancer progression in vivo.

### GPR87 overexpression activates the NF-κB signaling pathway in pancreatic cancer

**Fig. 5:**
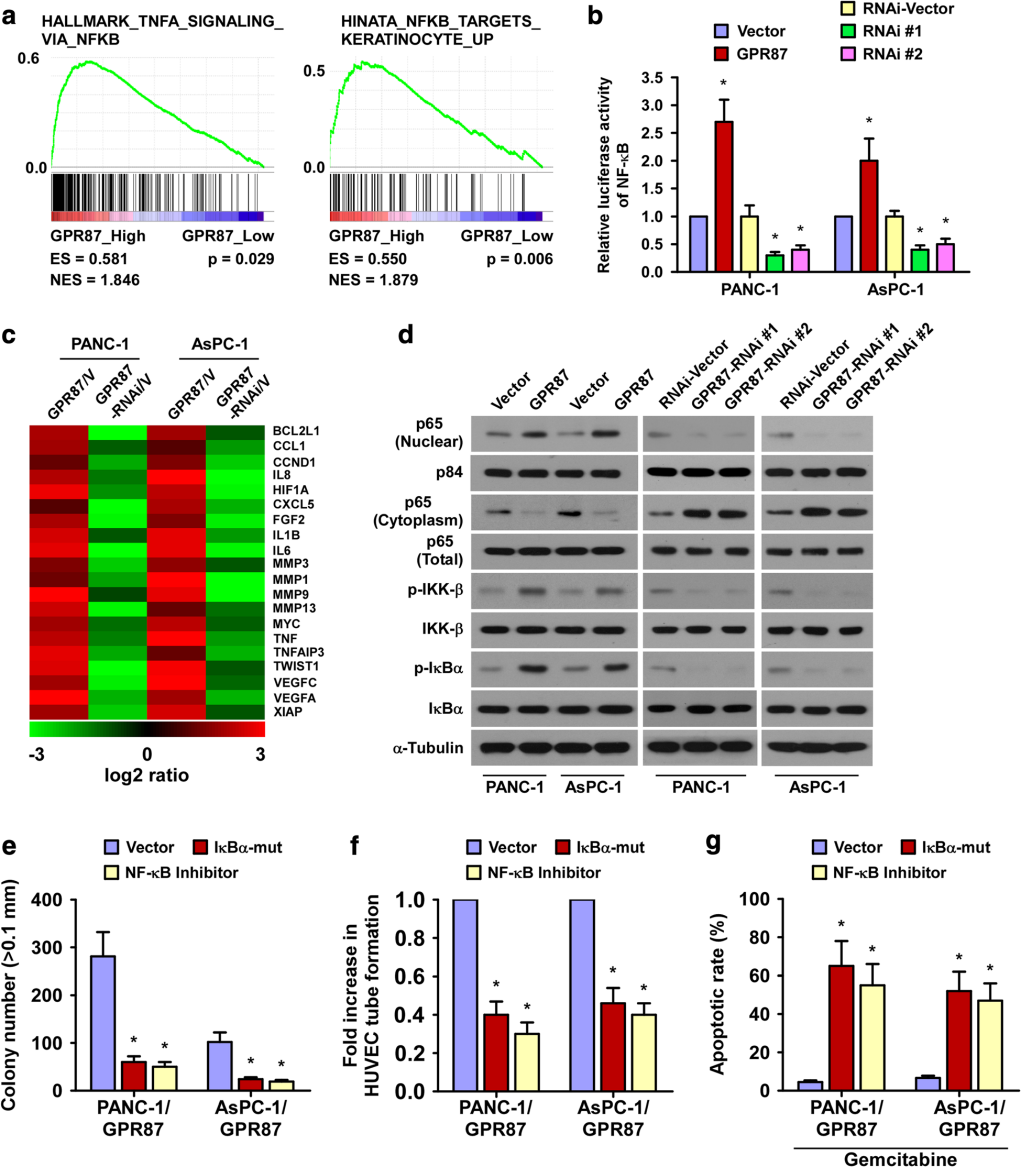
GPR87 up-regulation activates the NF-κB signaling pathway in pancreatic cancer. **a**. GSEA plots, demonstrating a significant correlation between the GPR87 mRNA expression levels in pancreatic cancer and the NF-κB-activated gene signatures from published datasets. **b**. Analysis of luciferase reporter activity in the indicated cells following transfection with 100 ng pNF-κB-luc plasmids or control-luciferase plasmid. **c**. Real-time PCR analysis demonstrating an apparent overlap between NF-κB-dependent gene expression and GPR87-regulated gene expression. The pseudo color represents an intensity scale for GPR87 versus vector or GPR87 siRNA versus control siRNA, calculated by log2 transformation. **d**. Western blotting analysis of the expression levels of the indicated proteins in the indicated cells. α-tubulin was used as a loading control. **e**. Quantification of colony numbers as determined by anchorage-independent growth assay. Colonies larger than 0.1 mm in diameter were scored. **f**. Quantification of tubule formation by HUVECs cultured in matrigel-coated plates with conditioned media from pancreatic cancer cells transfected with the vector, IκBα-mut or treated with the NF-κB inhibitor (JSH-23). **g**. Quantification of gemcitabine-induced (1 μM) TUNEL-positive cells in pancreatic cells transfected with vector, IκBα-mut or treated with the NF-κB inhibitor. Each bar represents the mean ± SD of three independent experiments. *p < 0.05

By analyzing GPR87 mRNA expression and NF-κB-regulated gene signatures from published pancreatic cancer patient profiles (TCGA datasets), we found that GPR87 expression was positively correlated with NF-κB signaling gene signatures (Fig. 5a and Additional file 6: Figure S4), raising the possibility that GPR87 upregulation may activate NF-κB signaling pathway in pancreatic cancer. In agreement with this supposition, NF-κB luciferase reporter activity was significantly increased in the GPR87-overexpressing cells but decreased in the GPR87-silenced cells (Fig. 5b). Moreover, the expressions of numerous well-known NF-κB downstream genes were shown to be elevated in GPR87-overexpressing cells but reduced in GPR87-silenced cells (Fig. 5c). Furthermore, western blotting revealed that the expression of nuclear p65 and phosphorylated-IKKβ and p-IκBα were significantly increased in GPR87-overexpressing cells but decreased in GPR87-silenced cells (Fig. 5d), suggesting that GPR87 promotes the activation of NF-κB signaling pathway.

To explore whether GPR87 mediates pancreatic cancer progression through NF-κB activation, luciferase reporter analysis was performed. Our results showed that the stimulatory effect of GPR87 on NF-κB activation was significantly inhibited by transfection of an IκBα dominant-negative mutant (IκBα-mut) or treatment with an NF-κB inhibitor. In addition, blockage of NF-κB signaling by either ectopically expressing IκBα-mu or treatment with NF-κB inhibitor drastically decreased colony numbers formed by GPR87-transduced cells in soft agar and reduced the capability of GPR87 overexpression induced invasiveness and angiogenesis (Fig. 5e-g). Furthermore, we found that the effects of NF-κB inhibitor in GPR87-overexpressing cells were much higher than that in naïve cells, and GPR87-RNAi treated cells only showed minimal response to NF-κB inhibitor (Fig. 5e-g and Additional file 7: Figure S5A-C). These results suggest that functional NF-κB activation is critical for GPR87-mediated aggressiveness of pancreatic cancer cells.

### Clinical relevance of GPR87-induced NF-κB activation in human pancreatic cancer

**Fig. 6:**
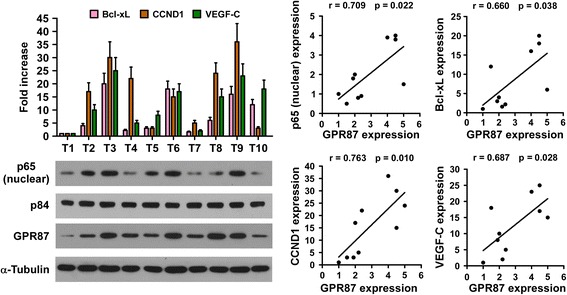
Clinical relevance of GPR87-induced NF-κB activation in human pancreatic cancer. Expression analysis (*left*) and correlation (*right*) of GPR87 expression and BCL-xL, CCND1, VEGF-C and nuclear p65 expression in 10 freshly collected human pancreatic tumor samples (T); α-Tubulin and the nuclear protein p84 were used as loading controls. Each bar represents the mean ± SD of three independent experiments

To further determine the clinical correlation between GPR87 and NF-κB signaling pathway, we examined whether GPR87 induced p65 nuclear accumulation in pancreatic cancer and elevated expression of NF-κB downstream genes in clinical samples. As shown in Fig. 6, GPR87 levels were positively correlated with nuclear p65 expression (*r* = 0.709, *p* = 0.022) and the mRNA expression levels of NF-κB downstream genes, Bcl-xL (*r* = 0.660, *p* = 0.038), CCND1 (*r* = 0.763, *p* = 0.010) and VEGF-C (*r* = 0.687, *p* = 0.028) in ten freshly collected clinical pancreatic cancer samples. Therefore, these results further support that GPR87 upregulation promotes the aggressiveness of pancreatic cancer and activates the NF-κB signaling pathway, which potentially leads to poor outcomes for patients with pancreatic cancer.

## Discussion

In the present study, we provided evidence for a novel link between GPR87 and the oncogenic NF-κB signaling pathway in pancreatic cancer. IHC analysis revealed that GPR87 was significantly upregulated in pancreatic cancer and correlated with clinical features and a poor prognosis of pancreatic cancer patients. Overexpression of GPR87 dramatically increased the anchorage-independent growth ability and the invasive capacity of pancreatic cells, promoted their ability to induce HUVEC tube formation and CAM neovascularization, and enhanced their resistance to apoptosis, while downregulation of GPR87 expression produced the opposite effects. These findings emphasize the prominent roles of oncogenic GPR87 in promoting carcinogenesis and progression of pancreatic cancer via NF-κB signaling pathway.

It is well established that the NF-κB signaling pathway not only regulates immune and inflammatory responses, but also plays an important role in oncogenesis [20, 21]. Therapeutic targeting of NF-κB signaling has been aggressively pursued for the treatment of a wide range of malignant pathologies in pancreatic cancer [5, 22–24]. Inhibition of NF-κB pathway by MG132, or sulfasalazine dramatically diminishes resistance to apoptosis induced by gemcitabine in multiple pancreatic cancer cell lines [5]. Additionally, NF-κB pathway inhibitor sodium salicylate inhibits phosphorylation and degradation of NF-κB negative regulator IκBα and subsequently enhances TNF-α induced apoptosis in BxPC-3 human pancreatic cancer cells [22]. Treatment with nafamostat mesilate, an NF-κB inhibitor, significantly decreases the volume and weight of nude mice xenografts derived from pancreatic cancer cells compared with the control group [23]. Furthermore, a nude mouse model was established and revealed that the number of liver metastases derived from pancreatic cancer cells was significantly decreased in the treatment group with the NF-κB inhibitor DHMEQ, compared with the control group [24]. Collectively, these findings provide a strong rationale for therapeutic targeting NF-κB signaling pathway in pancreatic cancer. Nevertheless, it is still a formidable challenge to archive improved treatment outcomes up to now. Although potential therapeutic approaches, such as the use of NF-κB or IKKβ inhibitors, could potentially exert anti-tumor effects on the cancer-promoting activities of NF-κB, they interfere with its various physiological functions in normal cells, such as functions in immunity and inflammation [21, 25]. Therefore, more effective therapeutic targets that regulate NF-κB signaling in an appropriate manner is warranted as an alternative to global NF-κB blockade. Herein, our study showed that GPR87 was overexpressed in pancreatic cancer and associated with poor survival of pancreatic cancer patients. Overexpressing GPR87 significantly promoted malignant phenotype including increased proliferation, angiogenesis, invasion and resistance to chemotherapeutic agent via impairing NF-κB signaling, while silencing GPR87 suppressed these effects. Meanwhile, we found that overexpressing GPR87 in HPDEC-1 dramatically increased the BrdU-positive cells and induced HUVEC tube formation (Additional file 8: Figure S6A-C). Furthermore, overexpression of GPR87 could also increase the proliferating rate of the esophageal squamous cell carcinoma (ESCC) cells (Additional file 8: Figure S6D-F), while silencing GPR87 produced the opposite effects, providing further evidence that GPR87 function as an onco-protein.

GPCRs represent the largest family of cell-surface molecules involved in signal transmission by far with more than 800 members, accounting for >2% of the total genes encoded by the human genome. These receptors regulate many cell functions, including cell proliferation, survival and motility, and have recently emerged as key players in processes contributing to tumor progression such as tumor growth, angiogenesis and metastasis [26]. GPR55, for example, has been reported to be upregulated in human squamous cell carcinomas and to enhance skin cancer cell anchorage-independent growth, invasiveness and tumorigenicity in vivo [27]. Overexpression of PAR1, a GPCR, promotes invasion and metastasis in breast cancer cells and inhibition of PAR1 signaling suppresses HMGA2-driven invasion in breast cancer cells [28]. P2Y2, a GPCR, is significantly overexpressed in prostate cancer and promotes cell invasion and metastasis both in vitro and in vivo [29]. Stevenson et al. reported that silencing the GPCR G-protein–coupled receptor galanin receptor 1 (GalR1) enhanced the effects of chemotherapy in drug-sensitive and resistant colorectal cancer cell lines [30]. Herein, we found that GPR87 was upregulated in pancreatic cancer and GPR87 overexpression promoted pancreatic tumor progression both in vivo and in vivo, which is in agreement with the oncogenic effects of GPR87 family members.

Accumulating evidence suggests that NF-κB signaling pathway can be activated by members of the GPCR family. Liu et al. reported that somatostatin type 2 receptor (SSTR2), a member of the GPCRs, activates NF-κB signaling pathway by leading to phosphorylation of IKKα/β and degradation of IκBα in pancreatic tumor cell line AR42J [31]. A previous study revealed that CXCL12 specifically signals through CXCR4, a member of the GPCRs to activate Akt and ERK and subsequently phosphorylate IκBα, causing activation of NF-κB signaling in pancreatic cancer cells [32]. Additionally, Lee and colleagues reported that activation of the BLT2 (a GPCR)-linked pathway leads to NOX-derived ROS generation and subsequent activation of NF-κB in prostate cancer cells [33]. Concordant with previous reports, our study showed that GPR87, a member of the GPCRs, could activate NF-κB signaling pathway in pancreatic cancer cells and that GPR87 levels were significantly associated with NF-κB activity in clinical pancreatic cancer tissues. However, the mechanism by which GPR87 activates NF-κB signaling pathway has yet to be elucidated and is the subject of current investigations by our group.

The mechanisms involved in GPR87 upregulation also remain to be characterized. According to TCGA [34], we found amplification of GPR87 in 25 of 184 cases, suggesting that amplification might account for GPR87 overexpression in 13.59% of pancreatic cancer patients. Interestingly, according to ChIP sequencing tracks in the University of California Santa Cruz (UCSC) genome browser (http://genome.ucsc.edu/cgi-bin/hgGateway) [35], the promoter region of GPR87 showed STAT3 binding elements, suggesting that tumor inflammatory microenvironment-mediated hyperactivation of JAK2/STAT3 signaling might contribute to GPR87 upregulation in pancreatic cancer. Thus, it would be of great interest to further investigate whether upregulation of GPR87 in pancreatic cancer is attributed to STAT3-mediated transcriptional upregulation. Apart from overexpression of GPR87, ligands, such as lysophosphatidic acid (LPA), in the tumor microenvironment may stimulate GPR87 and subsequently lead to constitutive activation of cancer-promoting signaling [36]. LPA is one of the most potent mitogens secreted in the tumor-associated environment including the ascites fluid produced by ovarian cancer cells, stimulating the LPA-sensitive GPCRs that are frequently overexpressed by these tumor cells and thus contributing growth, survival and resistance to chemotherapy [37]. Meanwhile, a recent study also showed that Gαi and Gαq contribute to GPR87-mediated NF-κB activation in the 293 T cells, which is ligand-independent [38], further support the notion that functional NF-κB activation is critical for GPR87-mediated cancer progression.

## Conclusion

In summary, we reported that GPR87 expression was significantly upregulated in pancreatic cancer, and higher GPR87 correlated with shorter overall survival of patients with pancreatic cancer. Overexpression of GPR87 promotes proliferation, metastasis, angiogenesis and resistance to apoptosis induced by a chemotherapeutic agent in pancreatic cancer. Our findings suggest that GPR87 plays a vital oncogenic role in pancreatic cancer progression and highlight its potential as a target for pancreatic cancer therapy.

## Methods

### Cell lines and cell culture

Human pancreatic cancer cell lines, including AsPC-1, Capan-1, BxPC-3, Capan-2, PANC-1 and MIA PaCa-2 were grown in the DMEM medium (Invitrogen) supplemented with 10% fetal bovine serum (HyClone). Primary cultures of normal human pancreatic ductal epithelial cells (HPDECs) were maintained in keratinocyte serum-free medium (KSFM; Invitrogen) with EGF (5 ng/ml) and BPE (50 μg/ml). All cells were incubated at 37 °C in a humidified atmosphere with 5% CO_2_.

### Patient information and tissue specimens

A total of 96 paraffin-embedded and archived pancreatic cancer samples, which were histopathologically and clinically diagnosed at the Union Hospital, Huazhong University of Science and Technology, from 2001.12 to 2009.01, were examined in this study. All the cases were recruited only based on positive diagnosis of pancreatic cancer without other additional criteria, and all the recruited cases were under standard treatment with chemotherapeutic agent gemcitabine. Survival time months correspond to time after diagnosis. Ten pairs of matched pancreatic cancer/adjacent noncancerous tissues and other 10 pancreatic cancer tissues were frozen and stored in liquid nitrogen until further use. Clinical information on the samples is summarized in Additional file 2: Table S1. The use of human tissue was approved by the local ethics committee (Tongji Medical College, China) and written informed consent was obtained from patients prior to surgery.

### Vectors, retroviral infection and transfection

The human GPR87 gene was PCR-amplified from cDNA and cloned into a pMSCV-puro retroviral vector and GPR87-targeting short hairpin RNA (shRNA) oligonucleotides sequences were cloned into pSuper-retro-puro to generate pSuper-retro-GPR87-RNAi(s). pNF-κB-luc and control plasmids (Clontech) were used to examine NF-κB activity. pBabe-puro-IκBα-mut (plasmid#15291) expressing IκBα dominant-negative mutant (IκBα-mut) was purchased from Addgene (Cambridge, MA). Transfection of siRNA or plasmids was performed using the Lipofectamine 3000 reagent (Invitrogen) according to the manufacturer’s instruction. Stable cell lines expressing GPR87 or GPR87 RNAi were selected for 10 days with 0.5ug/ml puromycin 48 h after infection.

### Western blotting analysis

Western blot was performed using anti-GPR87 (Abcam), anti-p-IκBα, IκBα and anti-p-IKKβ, IKKβ, anti-p65, anti-p84 antibodies (Cell Signaling Technology). The membranes were stripped and re-probed with an anti-α-tubulin antibody (Sigma) as a loading control.

### Immunohistochemistry (IHC)

IHC analysis was performed to study altered protein expression in 96 paraffin-embedded and archived pancreatic cancer with GPR87 antibody (Abcam, 1:500). The degree of immunostaining of formalin-fixed, paraffin-embedded sections were reviewed and scored separately by two independent pathologists uninformed of the histopathological features and patient data of the samples. The scores were determined by combining the proportion of positively-stained tumor cells and the intensity of staining. The scores given by the two independent pathologists were combined into a mean score for further comparative evaluation. Tumor cell proportions were scored as follows: 0, no positive tumor cells; 1, <10% positive tumor cells; 2, 10%–35% positive tumor cells; 3, 35–75% positive tumor cells; 4, >75% positive tumor cells. Staining intensity was graded according to the following standard: 1, no staining; 2, weak staining (light yellow); 3, moderate staining (yellow brown); 4, strong staining (brown). The staining index (SI) was calculated as the product of the staining intensity score and the proportion of positive tumor cells. Using this method of assessment, we evaluated protein expression in malignant lesions by determining the SI, with possible scores of 0, 2, 3, 4, 6, 8, 9, 12, and 16. Then the median value, SI = 8, was chosen as the cut off value, which samples with a SI ≥ 8 were determined as high expression and samples with a SI < 8 were determined as low expression.

### Invasion assay

Transwell inserts for 24-well plates (Corning Costar Corp., Cambridge, MA) were coated with prediluted Matrigel (BD Biosciences, Bedford, MA) and allowed to gel at 37 °C for 30 min. Cells were seeded at a density of 3 × 10^5^ per insert and the lower chamber of the Transwell was filled with 500 μL DMEM supplemented with 10% FBS. After 24 h of incubation, cells remaining on the upper surface of the Transwell membrane were removed by a cotton swab. Cells that had invaded through the Matrigel to the bottom of the insert were fixed, stained, photographed and quantified by counting them in 6 random high-powered fields.

### Chicken chorioallantoic membrane (CAM) assay

CAM assay was performed at day 6 of fertilized chicken eggs using a method previously described [39]. A 1.0-cm diameter window was opened on the egg shell (Huahao Breeding Co. Ltd, Hubei, China). The surface of the dermic sheet on the floor of the air sac was removed to expose the CAM. A 0.5-cm diameter filter paper was first placed on top of the CAM, and 100 μl conditioned medium was added onto the center of the paper. After the window was closed with sterile adhesive tape, the eggs were incubated at 37 °C under 80–90% relative humidity for 4 days. Following fixation with stationary solution (methanol: acetone = 1:1) for 15 min, the CAMs were cut and harvested, and gross photos of each CAM were taken with a digital camera (Panasonic, Osaka, Japan). The effect of conditioned media harvested from different cultured cells was evaluated by the number of second- and third-order vessels.

### HUVEC tube formation assay

Briefly, 200 μl of precooled Matrigel (Collaborative Biomedical Products) was transferred into each well of a 24-well plate and polymerized for 30 min at 37 °C. HUVECs (2 × 10^4^) in 200 μl of conditioned medium were added to each well and incubated at 37 °C, 5% CO_2_ for 20 h. The capillary tube structure was photographed under a 100 × bright-field microscope, and quantified by measuring the total length of the completed tubes. Each condition was assessed in triplicate.

### Animal studies, IHC, and H&E staining

BALB/c-nu mice (4–5 weeks of age, 18-20 g) were purchased from Vitalriver (Beijing, China). All experimental procedures were approved by the Ethics Committee of Tongji Medical College, Huazhong University of Science and Technology. The BALB/c nude mice were randomly divided into two groups (*n* = 6/group). One group of mice was inoculated subcutaneously with PANC-1/Vector cells (2 × 10^6^) in the left dorsal flank and with PANC-1/GPR87 cells (2 × 10^6^) in the right dorsal flank per mouse. Another group was inoculated subcutaneously with PANC-1/RNAi-vector cells (2 × 10^6^) in the left dorsal flank and with PANC-1/GPR87-RNAi cells (2 × 10^6^) in the right dorsal flank. Tumors were examined twice weekly; length and width measurements were obtained with calipers and tumor volumes were calculated using the equation (L × W^2^)/2. On day 36, tumors were detected by an IVIS imaging system, and animals were euthanized, tumors were excised, weighed and paraffin-embedded. Serial 6.0 μm sections were cut and subjected to IHC analyzed using an anti-Ki67 and anti-CD31 antibodies (Dako). Proliferation index was quantized by counting proportion of Ki67-positive cells. Apoptotic index was measured by percentage of TUNEL-positive cells.

### Microarray data process and analysis

The publicly-available gene expression dataset GSE16515 was assessed through the Gene Expression Omnibus (GEO) Datasets website http://www.ebi.ac.uk/arrayexpress/experiments/E-GEOD-16515/?query=gse16515. This array consists of 36 tumor samples and 16 normal samples; a total of 52 samples. 16 samples consist of both tumor and normal expression data, whereas 20 samples consist of only tumor data. This microarrays was used to identify the expression differences of genes between the pancreatic tumor and normal samples.

### Statistical analysis

Statistical tests for data analysis included Fisher’s exact test, log-rank test, Chi-square test, and Student’s 2-tailed *t* test. Statistical analyses were performed using the SPSS 11.0 statistical software package. Data represent mean ± SD. *p* < 0.05 was considered statistically significant.

## Additional files

Additional file 1: Figure S1. mRNA expression analysis shows that GPR87 was up-regulated in pancreatic cancer tissues and cell lines. A. Real-time PCR analysis of GPR87 expression in two human pancreatic ductal epithelial cells (HPDECs) and in pancreatic cancer cell lines (AsPC-1, Capan-1, BxPC-3, Capan-2, PANC-1 and MIA PaCa-2). B. Real-time PCR analysis of GPR87 expression in pancreatic cancer tissues (T) with matched adjacent non-tumor tissues (N) from 8 patients. Transcript levels were normalized to *GAPDH* expression. Each bar represents the mean ± SD of three independent experiments. * *p* < 0.05. (TIF 110 kb)
Additional file 2: Table S1. Clinicopathological characteristics of studied patients and expression of GPR87 in pancreatic cancer. (DOC 41 kb)
Additional file 3: Table S2. Correlation between the clinicopathological features and expression of GPR87. (DOC 41 kb)
Additional file 4: Figure S2. Annexin V-FITC and PI staining of indicated cells with or without Fluorouracil treatment. A. Representative images (left panel) and quantification (right panel) of Annexin V-FITC and PI staining of indicated cells with no treatment for 24 h. B. Representative images (left panel) and quantification (right panel) of Annexin V-FITC and PI staining of indicated cells treated with Fluorouracil for 24 h. Each bar represents the mean ± SD of three independent experiments. * *p* < 0.05. (TIF 508 kb)
Additional file 5: Figure S3. Western blot analysis of GPR87 expression in the indicated xenografts tumors. (TIF 166 kb)
Additional file 6: Figure S4. GPR87 up-regulation activates the NF-κB signaling pathway in pancreatic cancer. GSEA plot, indicating a significant correlation between the mRNA levels of GPR87 expression in pancreatic cancer and the NF-κB-activated gene signatures in multiple published datasets. (TIF 316 kb)
Additional file 7: Figure S5. Effects of Inhibiting NF-κB signaling in the indicated cells. A. Quantification of colony numbers as determined by anchorage-independent growth assay. Colonies larger than 0.1 mm in diameter were scored. B. Quantification of tubule formation by HUVECs cultured in matrigel-coated plates with conditioned media from pancreatic cancer cells transfected with the vector, IκBα-mut or treated with the NF-κB inhibitor (JSH-23). C. Quantification of gemcitabine-induced (1 μM) TUNEL-positive cells in pancreatic cells transfected with vector, IκBα-mut or treated with the NF-κB inhibitor. Each bar represents the mean ± SD of three independent experiments. * *p* < 0.05. (TIF 288 kb)
Additional file 8: Figure S6. Overexpressing GPR87 promotes proliferation and induces HUVEC tube formation in HPDEC cells and Eca109. A. Western blot of GPR87 expression in HPDEC-1 cells transfected with GPR87 or a vector control. α-tubulin was used as a loading control. B. Quantification of BrdU labeling in HPDEC-1 cells transfected with GPR87 or a vector control. C. Quantification of HUVECs cultured on matrigel-coated plates with conditioned medium from vector control or GPR87 transfected HPDEC-1 cells. D. Western blot of GPR87 expression in Eca109 cells transfected with GPR87, GPR87-RNAi or their corresponding controls. α-tubulin was used as a loading control. E. Quantification of BrdU labeling in indicated cells. F. Quantification of HUVECs cultured on matrigel-coated plates with conditioned medium from indicated cells. (TIF 396 kb)

## Abbreviations

CAM: Chicken chorioallantoic membrane
ESCC: Esophageal squamous cell carcinoma
GEO: Gene expression Omnibus
GPCR: G protein-coupled receptor
HPDECs: Human pancreatic ductal epithelial cells
HUVEC: Human umbilical vein endothelial cell
IHC: Immunohistochemistry
LPA: Lysophosphatidic acid
MVD: Microvascular density
NSCLC: Non-small cell lung carcinoma
TCGA: The cancer genome Atlas
UCSC: the University of California Santa Cruz (UCSC)
