# Supplementary figures and images for: Overexpression of G protein-coupled receptor GPR87 promotes pancreatic cancer aggressiveness and activates NF-κB signaling pathway
Source: Mol Cancer. 2017 Mar 14;16:61. doi: 10.1186/s12943-017-0627-6 (PMC5348802; doi:10.1186/s12943-017-0627-6)

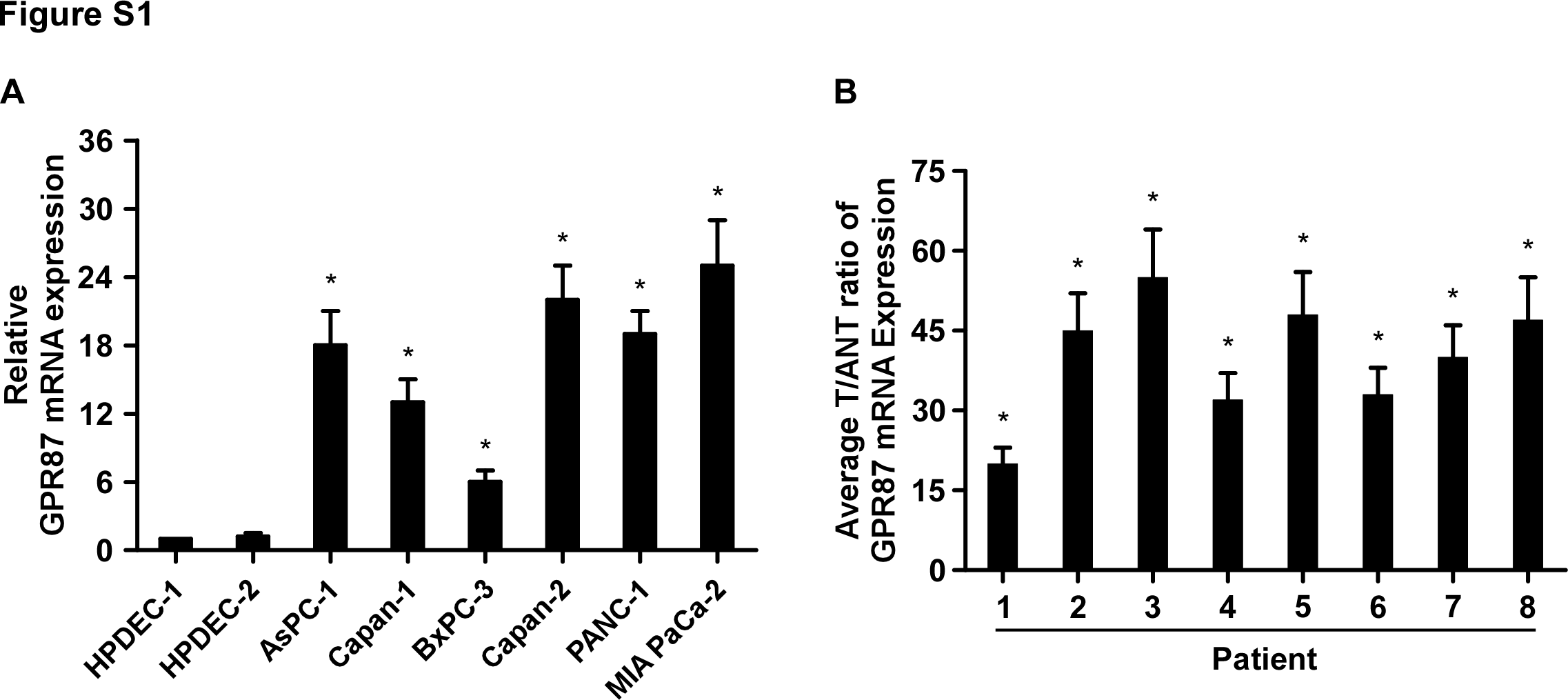

Supplement: Additional file 1: Figure S1. — mRNA expression analysis shows that GPR87 was up-regulated in pancreatic cancer tissues and cell lines. A. Real-time PCR analysis of GPR87 expression in two human pancreatic ductal epithelial cells (HPDECs) and in pancreatic cancer cell lines (AsPC-1, Capan-1, BxPC-3, Capan-2, PANC-1 and MIA PaCa-2). B. Real-time PCR analysis of GPR87 expression in pancreatic cancer tissues (T) with matched adjacent non-tumor tissues (N) from 8 patients. Transcript levels were normalized to GAPDH expression. Each bar represents the mean ± SD of three independent experiments. * p < 0.05. (TIF 110 kb) [file 12943_2017_627_MOESM1_ESM.tif]

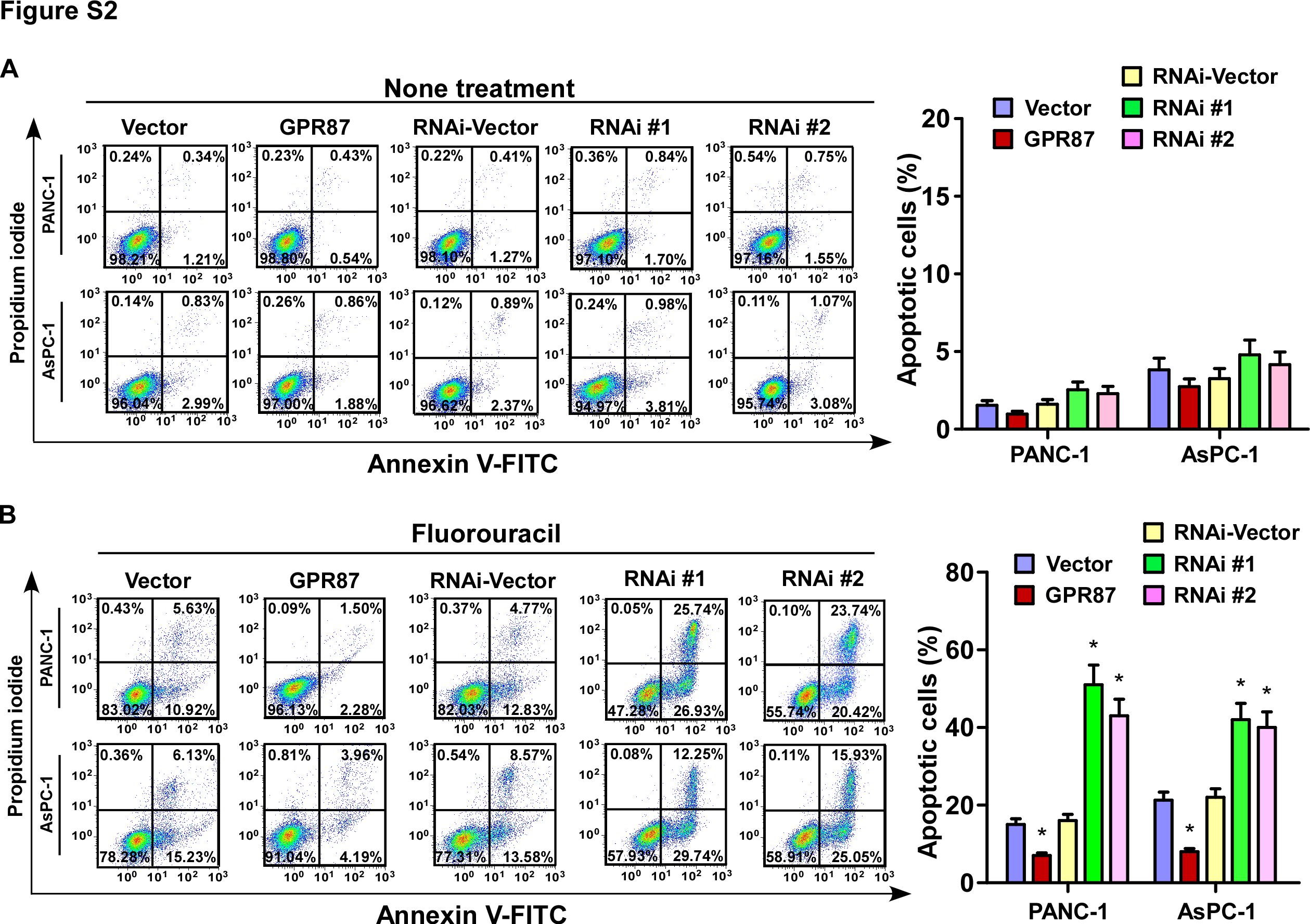

Supplement: Additional file 4: Figure S2. — Annexin V-FITC and PI staining of indicated cells with or without Fluorouracil treatment. A. Representative images (left panel) and quantification (right panel) of Annexin V-FITC and PI staining of indicated cells with no treatment for 24 h. B. Representative images (left panel) and quantification (right panel) of Annexin V-FITC and PI staining of indicated cells treated with Fluorouracil for 24 h. Each bar represents the mean ± SD of three independent experiments. * p < 0.05. (TIF 508 kb) [file 12943_2017_627_MOESM4_ESM.tif]

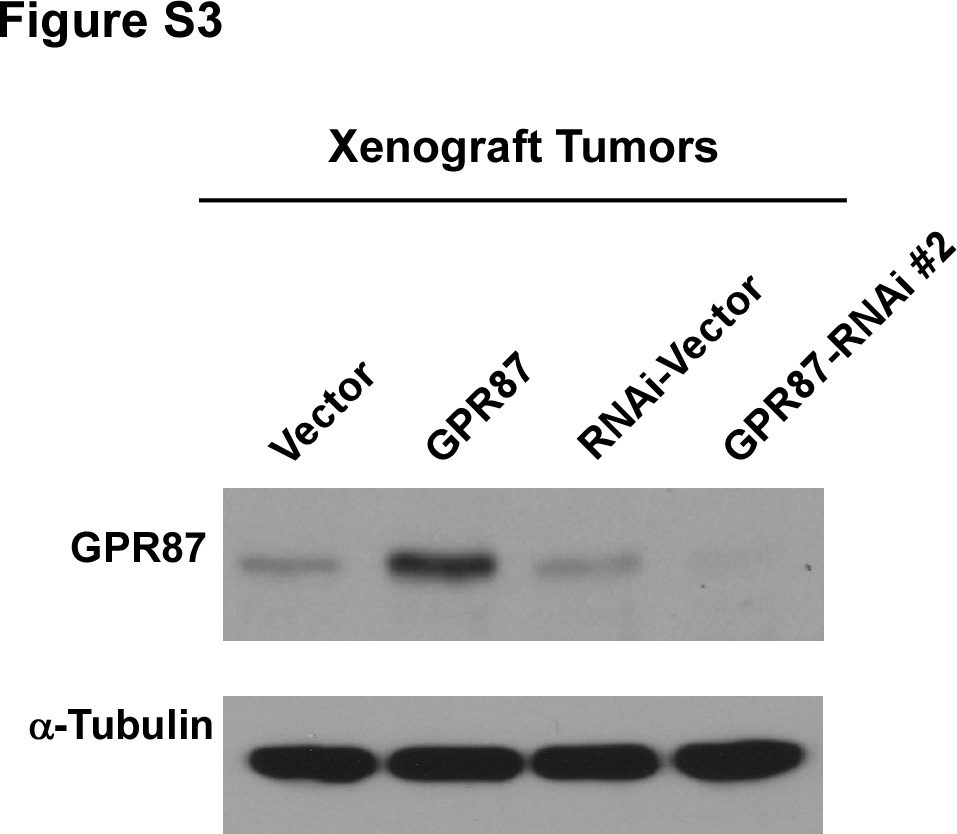

Supplement: Additional file 5: Figure S3. — Western blot analysis of GPR87 expression in the indicated xenografts tumors. (TIF 166 kb) [file 12943_2017_627_MOESM5_ESM.tif]

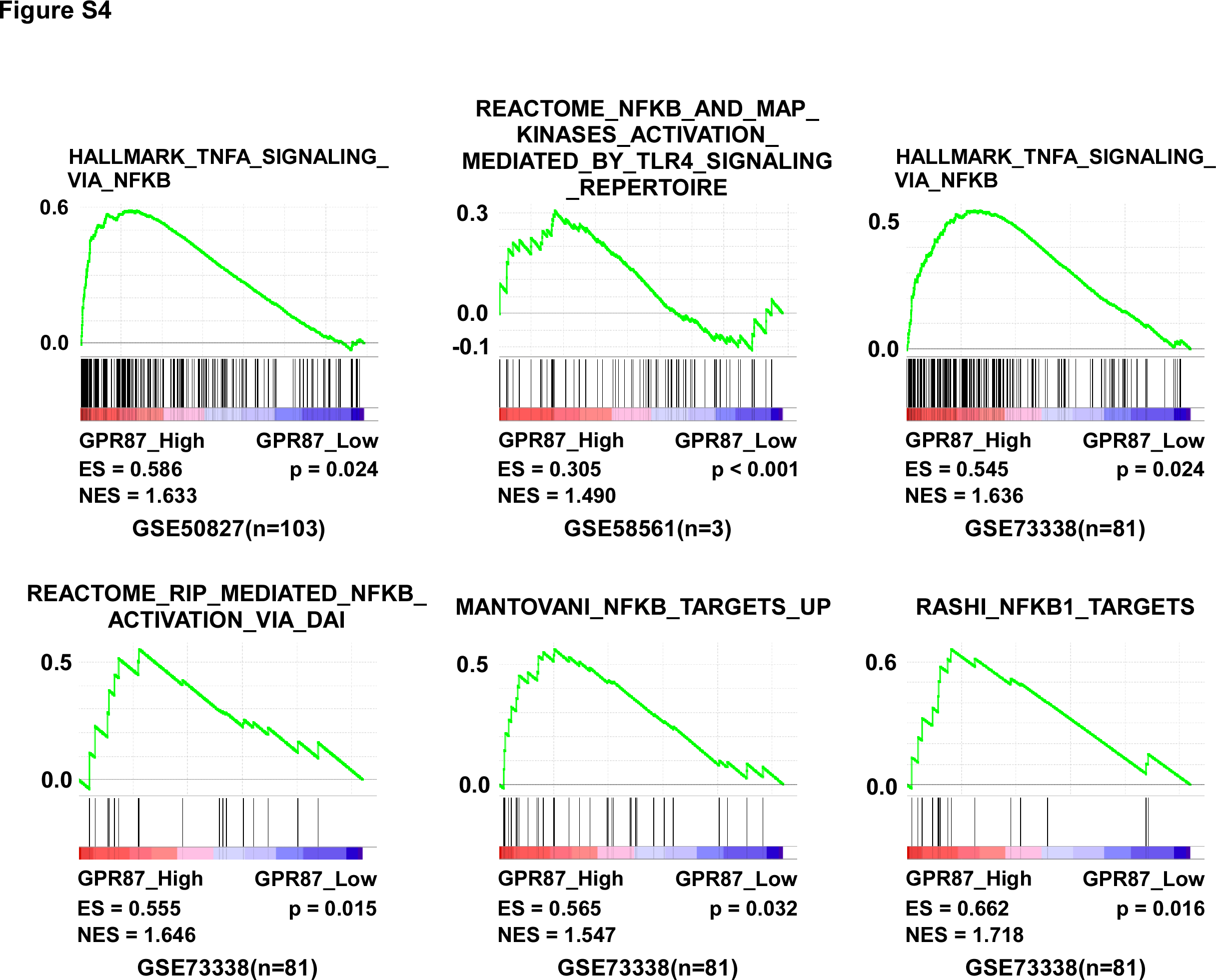

Supplement: Additional file 6: Figure S4. — GPR87 up-regulation activates the NF-κB signaling pathway in pancreatic cancer. GSEA plot, indicating a significant correlation between the mRNA levels of GPR87 expression in pancreatic cancer and the NF-κB-activated gene signatures in multiple published datasets. (TIF 316 kb) [file 12943_2017_627_MOESM6_ESM.tif]

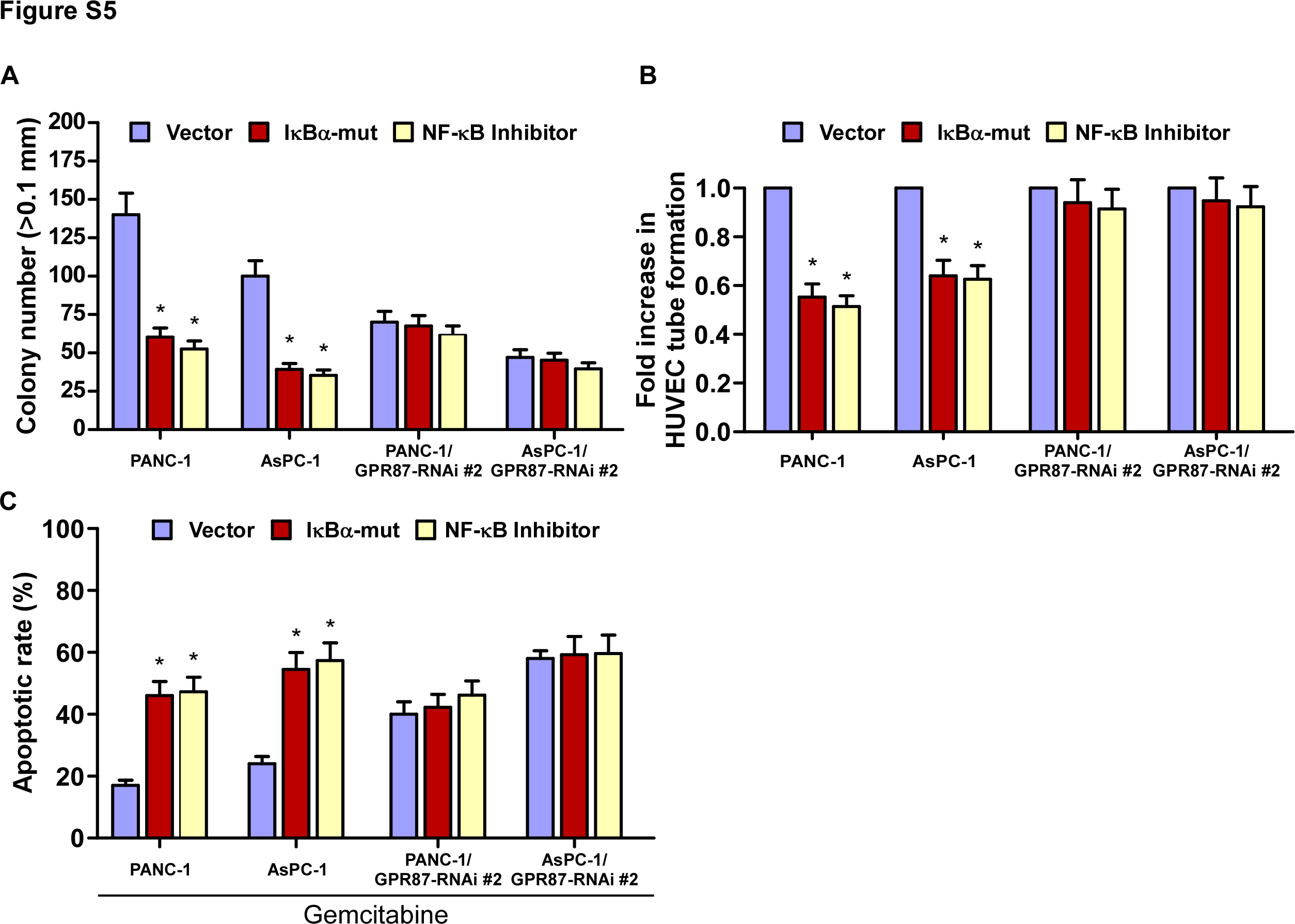

Supplement: Additional file 7: Figure S5. — Effects of Inhibiting NF-κB signaling in the indicated cells. A. Quantification of colony numbers as determined by anchorage-independent growth assay. Colonies larger than 0.1 mm in diameter were scored. B. Quantification of tubule formation by HUVECs cultured in matrigel-coated plates with conditioned media from pancreatic cancer cells transfected with the vector, IκBα-mut or treated with the NF-κB inhibitor (JSH-23). C. Quantification of gemcitabine-induced (1 μM) TUNEL-positive cells in pancreatic cells transfected with vector, IκBα-mut or treated with the NF-κB inhibitor. Each bar represents the mean ± SD of three independent experiments. * p < 0.05. (TIF 288 kb) [file 12943_2017_627_MOESM7_ESM.tif]

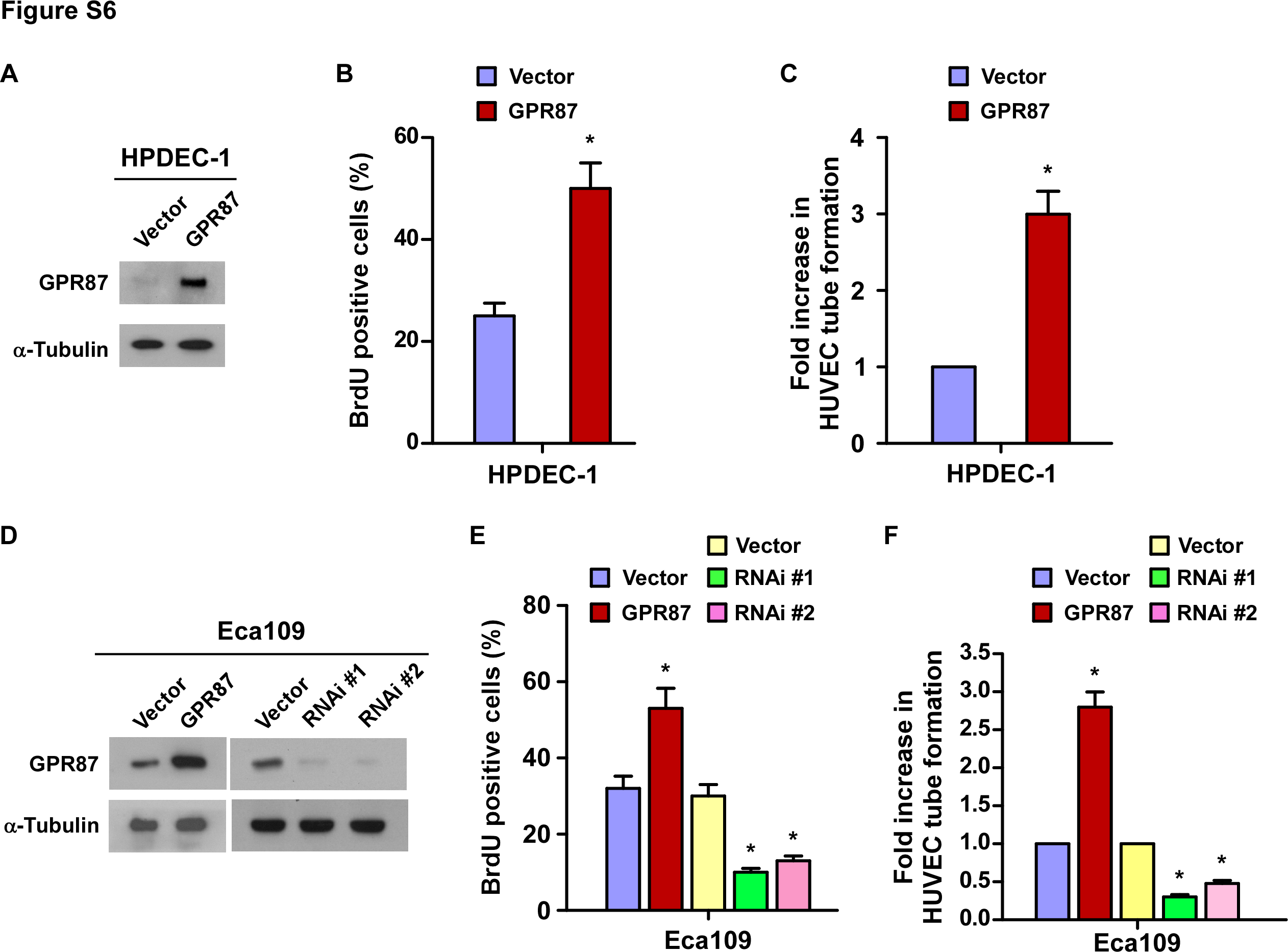

Supplement: Additional file 8: Figure S6. — Overexpressing GPR87 promotes proliferation and induces HUVEC tube formation in HPDEC cells and Eca109. A. Western blot of GPR87 expression in HPDEC-1 cells transfected with GPR87 or a vector control. α-tubulin was used as a loading control. B. Quantification of BrdU labeling in HPDEC-1 cells transfected with GPR87 or a vector control. C. Quantification of HUVECs cultured on matrigel-coated plates with conditioned medium from vector control or GPR87 transfected HPDEC-1 cells. D. Western blot of GPR87 expression in Eca109 cells transfected with GPR87, GPR87-RNAi or their corresponding controls. α-tubulin was used as a loading control. E. Quantification of BrdU labeling in indicated cells. F. Quantification of HUVECs cultured on matrigel-coated plates with conditioned medium from indicated cells. (TIF 396 kb) [file 12943_2017_627_MOESM8_ESM.tif]
