# Supplementary material for: Overexpression of G protein-coupled receptor GPR87 promotes pancreatic cancer aggressiveness and activates NF-κB signaling pathway
Source: Mol Cancer. 2017 Mar 14;16:61. doi: 10.1186/s12943-017-0627-6 (PMC5348802; doi:10.1186/s12943-017-0627-6)
Supplement: Additional file 2: Table S1. — Clinicopathological characteristics of studied patients and expression of GPR87 in pancreatic cancer. (DOC 41 kb) [file 12943_2017_627_MOESM2_ESM.doc]

**Additional file 2: Table S1**. Clinicopathological characteristics of studied patients and expression of GPR87 in pancreatic cancer

| **Factor** | **No.** | **(%)** |
| --- | --- | --- |
| **Gender** |  |  |
| Male | 44 | 45.8 |
| Female | 52 | 54.2 |
| **Age (years)** |  |  |
| ≤65 | 49 | 51.0 |
| >65 | 47 | 49.0 |
| **Clinical stage** |  |  |
| I | 13 | 13.5 |
| II | 30 | 31.3 |
| III | 41 | 42.7 |
| IV | 12 | 12.5 |
| **T classification** |  |  |
| T_1_ | 10 | 10.4 |
| T_2_ | 34 | 35.4 |
| T_3_ | 45 | 46.9 |
| T_4_ | 7 | 7.3 |
| **N classification** |  |  |
| N_0_ | 70 | 72.9 |
| N_1_ | 26 | 27.1 |
| **M classification** |  |  |
| No | 64 | 66.7 |
| Yes | 32 | 33.3 |
| **Vital status** |  |  |
| Alive | 31 | 32.2 |
| Dead | 65 | 67.8 |
| **Expression of GPR87** |  |  |
| Low expression | 47 | 49.0 |
| High expression | 49 | 51.0 |
