# Supplementary material for: Overexpression of G protein-coupled receptor GPR87 promotes pancreatic cancer aggressiveness and activates NF-κB signaling pathway
Source: Mol Cancer. 2017 Mar 14;16:61. doi: 10.1186/s12943-017-0627-6 (PMC5348802; doi:10.1186/s12943-017-0627-6)
Supplement: Additional file 3: Table S2. — Correlation between the clinicopathological features and expression of GPR87. (DOC 41 kb) [file 12943_2017_627_MOESM3_ESM.doc]

**Additional file 3: Table S2**. Correlation between the clinicopathological features and expression of GPR87

| **Patient characteristics** | | **GPR87 expression**  **(IHC Score)** | | ***P*-value** |
| --- | --- | --- | --- | --- |
|  |  | **Low or none** | **High** |  |
| **Gender** | Male | 19 | 25 | 0.314 |
|  | Female | 28 | 24 |  |
| **Age (years)** | ≤65 | 22 | 27 | 0.540 |
|  | >65 | 25 | 22 |  |
| **Clinical stage** | I | 9  132  62 | 4  44 | 0.011 |
|  | II | 20 | 10 |  |
|  | III | 15 | 26 |  |
|  | IV | 3 | 9 |  |
| **T classification** | T_1_ | 7  132  62 | 3  44 | 0.002 |
|  | T_2_ | 24 | 10 |  |
|  | T_3_ | 14 | 31 |  |
|  | T_4_ | 2 | 5 |  |
| **N classification** | N_0_ | 40  204 | 30  0 | 0.011 |
|  | N_1_ | 7 | 19 |  |
| **M classification** | No | 37 | 27 | 0.018 |
|  | Yes | 10 | 22 |  |
| **Vital status** | Alive | 18 | 13 | < 0.001 |
|  | Dead | 29 | 36 |  |
